# Photocatalytic Material-Microorganism Hybrid System and Its Application—A Review

**DOI:** 10.3390/mi13060861

**Published:** 2022-05-30

**Authors:** Jiaao Song, Huichao Lin, Gaozhen Zhao, Xiaowen Huang

**Affiliations:** State Key Laboratory of Biobased Material and Green Papermaking, Department of Bioengineering, Qilu University of Technology (Shandong Academy of Sciences), Jinan 250300, China; songjiaao0419@126.com (J.S.); linhuichao2020@gmail.com (H.L.); zhaogaozhen2021@126.com (G.Z.)

**Keywords:** hybrid photocatalysis, synthetic biohybrid, photoenzymatic synthesis

## Abstract

The photocatalytic material-microorganism hybrid system is an interdisciplinary research field. It has the potential to synthesize various biocompounds by using solar energy, which brings new hope for sustainable green energy development. Many valuable reviews have been published in this field. However, few reviews have comprehensively summarized the combination methods of various photocatalytic materials and microorganisms. In this critical review, we classified the biohybrid designs of photocatalytic materials and microorganisms, and we summarized the advantages and disadvantages of various photocatalytic material/microorganism combination systems. Moreover, we introduced their possible applications, future challenges, and an outlook for future developments.

## 1. Introduction

With the growth of population and economy, as well as the improvement of living standards, the consumption of energy rises sharply. Alongside the continuous consumption of non-renewable energy sources, resulting problems such as environmental pollution and food shortages are becoming more severe, making green and renewable energy technology development a global research priority [1,2,3]. One hour of solar energy is equivalent to the entire amount of energy that is used by humanity in a year [4,5]. Hence, the conversion, transportation, and storage of solar energy is of significant concern to scientists [6]. Natural photosynthesis is the most common mechanism of solar energy conversion. Plants, algae, and photosynthetic bacteria can absorb solar energy into chemical energy [7,8,9,10]. However, the efficiency of natural photosynthesis is quickly saturated. Excessive sunlight can lead to photodamage. Repairing photodamage requires energy consumption, resulting in an even lower conversion of solar energy [11,12]. The conversion rate only reaches 7%, and the storage efficiency does not exceed 1% under optimal conditions [13,14,15]. Artificial photosynthesis mimics natural photosynthesis by using semiconductor photocatalytic materials to capture light energy. It has greater designability and a more comprehensive range of wavelength utilization, but it does not have the selective catalytic capabilities. The combination of artificial photosynthesis and natural biocatalytic systems efficiently creates high value-added products. This approach can improve solar energy conversion and storage, and so biocatalysis and artificial component catalysis cannot be performed independently [16,17]. Existing systems, in which photocatalysts such as TiO_2_ [18], CdS [19], and g-C_3_N_4_ [20] can be combined with specific microorganisms to form heterogeneous systems, have become a research hotspot. In this review, we mainly summarize the construction methods and application fields of hybrid systems, discuss the challenges and opportunities for their future development, and provide references for researchers in this field.

## 2. Photocatalytic Materials—Microbial Hybrid System

The hybrid photocatalytic material-microorganism system combines the strength of photocatalytic material and microorganisms. The self-replication, self-healing, and intracellular metabolic pathways of microbial cells provide potential stability and scalability to cell-based systems [21]. Photocatalytic materials act as light absorbers and generate photogenerated electrons. Microorganisms use these electrons to participate in metabolic pathways for the targeted synthesis of complex products [22]. Nowadays, semiconductors, dyes/polymers, and electrodes can be combined with microorganisms to prepare hybrid photocatalytic material-microorganism systems (Table 1) [23,24].

### 2.1. Semiconductor Material-Microorganism Hybrid System

**Figure 1:**
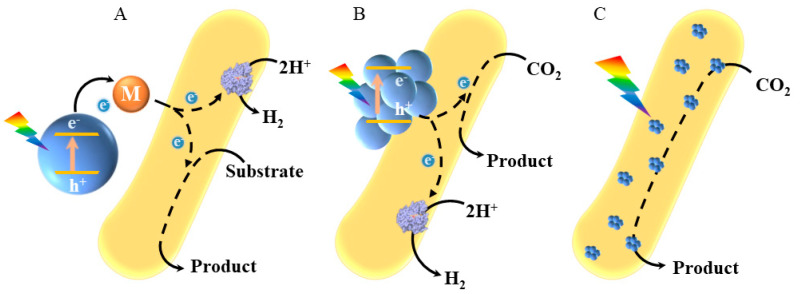
In material-microbe hybrids, the material can be distributed outside the cell. (**A**) On the cell membrane; (**B**) Inside the cell; (**C**) The photogenerated electrons generated by the material will enter the microbial cell that supplies energy for intracellular metabolism.

In semiconductor material-microbe hybrid systems, semiconductor materials are commonly distributed outside of the microbial cell, on the cell membrane, and inside the cell (Figure 1). Light excites the semiconductor material to generate electron-hole pairs. Photogenerated electrons are then transferred to the microorganism to provide energy for intracellular metabolic pathways, and the target products are selectively synthesized.

When the material is extracellular, it needs to use an electronic medium to transfer electrons. Honda et al. developed a hybrid system of TiO_2_, methylviologen (MV), and recombinant *E. coli* to realize highly efficient light-driven H_2_ production mediated via MV [46]. Rowe et al. gained access to a varied and selective set of visible light-driven chemical syntheses without enzyme purification, via the use of MV to transfer photoenergized electrons to the corresponding enzymes in *Shewanella oneidensis*. However, MV is toxic and poorly biocompatible, leading to cell death after the reaction [47]. Photogenerated electrons cross the cell membrane to participate in intracellular metabolic pathways when the material is on the cell surface. In this field, the research results of Yang’s team are of milestone significance. To enable it to function as a light harvester, they precipitated cadmium sulfide nanoparticles on the surface of a nonphotosynthetic bacteria. The collected energy fueled cellular metabolism, resulting in acetic acid production [25]. Guo et al. attached light-harvesting indium phosphide nanoparticles (InP) to the surface of *Saccharomyces cerevisiae* (*S. cerevisiae*). Photogenerated electrons pass through the cell membrane to drive the reduction of cellular NADPH in yeast, which can facilitate the production of shikimic acid [28]. The hybrid system, which was composed of *Moorella thermoacetica*-CdS + TiO_2_-MnPc, adopted the Z scheme strategy, in which MnPc selectively reduces cysteine (CySS) with a higher redox efficiency and a longer acetic acid synthesis time [48]. When the material is inside the cell, photogenerated electrons are generated intracellularly, and they are directly involved in intracellular metabolic pathways. Yang’s group replaced cadmium sulfide (CdS) nanoparticles with gold nanoclusters (Au NCs), which had a better biocompatibility and a smaller size. Au NCs could enter the cell, and they further improved the charge transfer rate of the photosensitizer, such that it could achieve a 33% increase in quantum efficiency for continuous CO_2_ fixation [26]. CdS NPs as photocatalytic materials could be used in addition to *M. thermoaccia*, and microorganisms such as *M. barkeri* [49], *R. palustris* [50], *T. denitrificans, T. thioparus* [51,52], *G. thioreducens* [32], and *D. desulfuricans* [53] could construct hybrid systems to achieve solar energy conversion.

Based on the above studies, we found that the charge transfer between photocatalytic materials and microorganisms is essential for studying heterogeneous systems. The electron transfer mode and its efficiency in different distribution systems was not well studied, and the conversion efficiency and the value of the final product were relatively low.

### 2.2. Dye/Polymer-Microorganism Hybrid System

Dye and polymer are also a class of photosensitizers that provide photogenerated electrons, and they can be used in the form of free molecules. It can compound with cells by electrostatic force, van der Waals force, or physical action, which facilitates the formation of well-contacted hybrids. Park’s group used eosin Y (EY) as a photosensitizer to enter the *E. coli* cell membrane and it was bound specifically to the heme structural domain of P450. In the absence of cofactors and redox partners, the photoactivated P450 catalytic cycle was realized [54]. Wang Shu’s group has developed a series of polymer photosensitive materials. They have designed a hybrid system through the electrostatic recombination of a photoactive cationic poly(fluorene-co-phenylene) derivative (PFP) and cyanobacterium (*Synechococcus* sp. PCC7942, *Syne*). PFP increased the efficiency of light utility and electron transport rate by amplifying the absorption zone of *Syne* [36]. Gai et al. coated perylene diimide derivatives (PDIs) and poly(fluorene-co-phenylene) (PFP) as photosensitizers onto the bacterial surface to form a PN heterojunction (PFP/PDI) layer, providing higher hole/electron separation efficiency, and fixed CO_2_ to synthesize acetic acid through the Wood-Ljundahl pathway [37]. Zhou et al. identified synthetic light-capturing polymers with green light absorption, and found that far-red emission poly(boron-dipyrromethene-co-fluorene) (PBF) could enhance the PSI activity of the alga *Chlorella vulgaris*, which further upgraded and enhanced the PSII activity of natural photosynthesis, and increased oxygen, ATP, and NADPH production [35]. From Qi et al., bio-palladium catalysts were found to synthesize photoactive polystyrene (PPE) on the surface of *C. pyrenoidosa* cells. It could expand light absorption and accelerate the cyclic electron transport, thus increasing the synthesis of ATP [38].

There are relatively few studies on dye/polymer-microorganism hybrid systems. The main reason for this may be that compared to solid semiconductor materials, dyes are firstly prone to photobleaching, challenging to adjust, and provide a weak reduction potential. Secondly, the energy bands of polymeric photosensitizers are regulated by functional group modifications and controlled polymerization. However, the products are mixtures and they have poor reproducibility. Finally, the dye/polymer photosensitizer is poorly biocompatible and environmentally toxic.

### 2.3. Electrode-Microbial Hybrid System

In the electrode-microbe hybrid system, the photoelectrodes provide electrons, H_2_, or redox mediators as cytoreductive equivalents that are transferred to cellular metabolic pathways for chemical transformation. The electrode-microbial hybrid system has better adjustability and operability. The systems can be divided into integrated and decentralized systems according to the interaction between the electrodes and the microorganisms.

**Figure 2:**
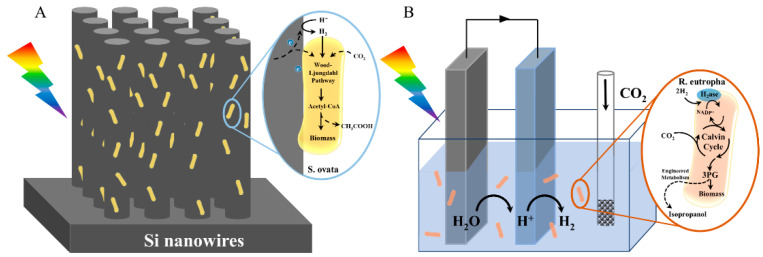
Typical samples of integrated and dispersed systems for electrode-microbial hybrid systems. (**A**) Integrated system. Construction of a hybrid system using tightly packed silicon nanowires and *S. ovata* to achieve a 3.6% solar energy conversion efficiency in 1 week. (© 2020 Elsevier Inc.) (**B**) Decentralized system. Wild-type and engineered NiMoZn hybrid systems generate biomass and isopropanol, respectively.

The integrated system requires the electrode to be in close contact with the microorganisms, and the electrons generated by the electrodes are directly transferred to the organisms [55,56]. A representative study is that Yang’s research group proposed the use of no light, microorganisms and silicon nanoarrays as cathodes, and titanium dioxide nanoarrays as anodes. The nanoarrays act as a light collector, which provides a large surface area, and then acetic acid is synthesized using the Wood-Ljungdahl pathway to fix CO_2_ without an auxiliary medium [39]. Due to insufficient interaction between the microorganisms and the electrodes, the applied overpotential and reduction efficiency of CO_2_ was relatively low. To improve this, Yang’s group adjusted the pH value of the electrolyte to increase buffering capacity, then fabricated a closely packed nanowire—*S. ovata* cathode formation that enhanced the reduction of CO_2_, with an electric current density of 0.65 mA cm^−2^ (Figure 2A) [40]. Chen et al. created a 3D-printed library of micro-pillar arrays of electrodes with different heights and surface features, and investigated the energy/electron transfer process across the bioelectrode interface, which was ultimately almost twice the photocurrent of an advanced porous structure with the same height [57].

The decentralized system involves the insertion of an electrode into a microbial suspension. The electrodes’ electrons are required to couple with hydrogen or the redox medium before the microorganisms can use them. Nichols et al. constructed a hybrid system with TiO_2_ as the photoanode and p-InP/Pt as the photocathode, compounded with *Methanosarcina barkeri*, using H_2_ as the reduction equivalent, to convert CO_2_ to CH_4_ with a total Faraday efficiency of up to 86% [58]. Nocera’s group constructed an electrode hybrid system by combining *R. eutropha* with a cobalt phosphate (CoPi) anode and a nickel-molybdenum-zinc (NiMoZn) cathode to convert CO_2_ into biomass and fusel efficiently (Figure 2B) [42]. However, the O_2_ generated by the anode accumulated active oxygen radicals in the system, and the electrode was easily corroded and released toxic metal ions during the reaction process. Based on this, Liu et al. used CoPi as the anode and Co-P as the cathode, and compounded this with *Raistonia eutropha*. The H_2_ produced by the cathode promoted CO_2_ reduction to synthesize biomass, fuel, or other chemical products [43]. In addition to carbon sequestration, his group used the aforementioned with an electrode combination of *Xanthobacter autotrophicus* to fix atmospheric nitrogen in NH_3_ or soluble biomass with high flux and energy efficiency [41]. The low dissolution rate of H_2_ in aqueous solutions limited the energy transfer efficiency. Rodrigues, R.M. et al. used biocompatible perfluorocarbon nanoemulsion as H_2_ carriers. Both the available H_2_ concentration and the efficiency of acetic acid synthesis were improved [44].

The slow charge transfer efficiency limits the conversion efficiency of the electrode-microbe hybrid system within the interface between the photoelectrode and the bacteria. The conversion efficiency and the product values remain low, even if the reaction possesses a high degree of selectivity. Although increasing the light intensity can effectively improve electron transfer efficiency, it adversely affects microorganisms and electrodes. Consequently, research on the chemical stability, biocompatibility, electrical conductivity, and surface chemistry of microorganisms that have been integrated into the electrode needs to be enhanced.

## 3. Application of the Photocatalytic Material-Microbe Hybrid System

### 3.1. Synthesis of Green Energy

With rapid modernization and the rise in energy consumption, the burning of fossil energy generates a large amount of greenhouse gases such as CO_2_. Thus, green energy is receiving increasing attention. The use of microorganisms to fix solar energy to generate green fuels not only solves the problems of non-renewable fossil fuels and environmental pollution, but also provides the possibility for establishing a clean and sustainable energy conversion and storage platform [59]. In recent years, a hybrid system constructed with photocatalytic materials and microorganisms has been significantly able to enhance the utilization efficiency of solar energy by microorganisms. The sustainable conversion of CO_2_ into fuel is considered to be an effective method for mitigating climate change and alleviating energy depletion [60,61,62,63,64].

**Figure 3:**
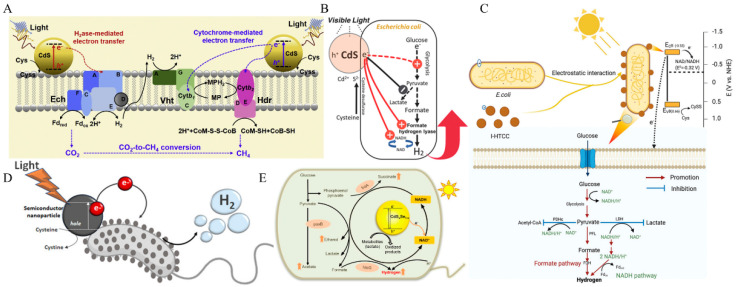
(**A**) Construction of *M. barkeri*-CdS biohybrid hybrid system could convert CO_2_ to CH_4_. (© 2019 Elsevier B.V. All rights reserved). (**B**) Precipitation of CdS nanoparticles on the surface of *E. coli* to improve biohydrogen production efficiency (© 2017 WILEY-VCH Verlag GmbH & Co. KGaA, Weinheim). (**C**) Construction of an I-HTCC@*Escherichia coli* biomixing system to promote hydrogen production (© 2021 Wiley-VCH GmbH). (**D**) Construction of a biohybrid system with *D. desulfuricans*-CdS nanoparticles to measure high H_2_ production activity (© 2021 Wiley-VCH GmbH). (**E**) Construction of an inorganic-biological hybrid CdSe_x_S_1-x_ nanoparticle for fermentation hydrogen production (© 2021 Elsevier B.V. All rights reserved).

Sakimoto et al. used a semiconductor light collector and *M. barkeri* to construct a hybrid system that reduced CO_2_ to CH_4_ [65]. Ye et al. developed a biological hybrid composed of CdS nanoparticles and non-phototrophic methanogens to convert CO_2_ directly to CH_4_, with a production efficiency of 0.19 μmol/h and a QE of 0.34% (Figure 3A) [49]. Xiao et al. presented a visible light-responding photochemical system for microorganisms that could reduce CO_2_ to CH_4_ [45]. The biological production of H_2_ possesses the advantages of raw material availability, self-sustainable, and system reproducibility [24,66]. Tang’s team initiated the in situ deposition of silica on the surface of *Chlorella* cells to form the core-shell structure of green algal aggregates. Although cell proliferation leads to the destruction of the aggregate structure, this structure has laid the experimental foundation for developing the field of biohydrogen production [67]. Wang et al. constructed a micro-aggregate with a positively charged polymer and negatively charged *Chlorella pyrenoidosa* (CP). The hydrogen production was continuous for 42 h, with an average hydrogen production rate of 0.26 μmol/mg chlorophyll per hour [68]. Wang’s group formed a hybrid system of *Escherichia coli* and CdS nanoparticles to produce H_2_ under anaerobic conditions (Figure 3B) [29]. Using this foundation, Wei et al. presented that H_2_ can be produced continuously for 96 h under natural aerobic conditions, by introducing a biomimetic silica encapsulation strategy in *E. coli* engineered bacteria [30]. Xiao et al. bound *E. coli* to negatively charged iodine-doped hydrothermal carbon (I-HTCC). A hybrid system was formed via electrostatic interaction, and the hydrogen production efficiency of 2000 W m^−2^ was 57.04% higher than that of pure *E. coli* (Figure 3C) [31]. Wang’s group used *Desulfovibrio desulfuricans* and CdS nanoparticles to construct a hybrid system with an H_2_ yield of 36 μmol/g_dcw_/h, which achieved continuous H_2_ generation for over 10 days (Figure 3D) [53]. Cui et al. self-assembled CdSex S 1-x semiconductor nanoparticles in *E. coli* to construct a hybrid system, in which the hydrogen yield was 2.6-fold greater than using extracellular nanoparticles (Figure 3E) [69]. Jiang et al and Wang et al. used AgInS_2_/In_2_S_3_ and CdSeS@ZnS quantum dots to combine with the surface of *E. coli* to produce H_2_ [33,70]. Chen et al. constructed a self-healing, sustainable, and low-cost photocatalytic material-microbe hybrid system with CdS nanoparticles and *Thiobacillus denitrificans*. The proportion of N_2_O in the product exceeded 96.4%, and the N_2_O yield reached 8.7 mg/L after 68 h of illumination [51].

### 3.2. Synthesis of High Value-Added Products

The photocatalytic material-microbial hybrid system demonstrates the virtues of high catalytic selectivity, mild reaction conditions, low energy consumption, and environmental friendliness in synthesizing high value-added products. The hybrid system has been developed as a novel and efficient platform for biomanufacturing, and it has been used to produce acetic acid, shikimic acid, and bioplastics [25,28,37,48,71,72,73].

**Figure 4:**
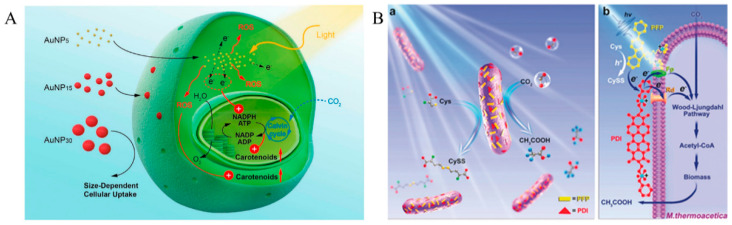
(**A**) *C. zofingiensis*-AuNPs hybrid system, where AuNPs significantly increase the relative electron transport rate in photosystem II, and the ROS level in microalgae (Copyright © 2020, American Chemical Society). (**B**) (**a**) PDI/PFP/*M. thermoacetica* photosynthesis hybrid system. (**b**) Transport pathway of photogenerated electrons generated by PDI/PFP in light (© 2020 Wiley-VCH Verlag GmbH & Co. KGaA, Weinheim).

A series of serious environmental problems are caused by massive CO_2_ emissions [74]. The hybrid systems can fix CO_2_ to produce high value-added products, which is a sustainable production strategy [75]. Askimoto et al. constructed a hybrid system with *M. thermoacetica* and CdS, and 90% of CO_2_ was converted to the target product, acetic acid; a maximum acetic acid production over 12 h could reach 1400 μmol/L, and the quantum yield reached 85% [48]. To further improve the yield of acetic acid, Yang’s group introduced biocompatible intracellular photocatalyst AuNCs in the system to replace CdS [26]. After adding TiO_2_, the acetic acid yield was increased by approximately 85%. Following four days of system operation, the biocompatible AuNCs improved the cumulative acetic acid yield by 14%. In addition to inorganic semiconductor materials, organic semiconductor materials are widely used in biosensing, gene/drug delivery, and other fields, due to their excellent biocompatibilities [76,77]. Li et al. combined *C. zofingiensis* with highly efficient light-trapping Au NPs to construct a hybrid system in which smaller Au NPs could enter the interior of *C. zofingiensis*. It promoted a carotenoid production of 10.7 ± 1.2 mg/L, which was 42.7% higher than that of natural microalgae (Figure 4A) [27]. Gai et al. constructed a hybrid system with an *M. thermoacetica* and PDI/PFP p-n heterojunction. The organic semiconductor PDI/PFP had excellent biocompatibility, and the number of surviving cells increased to approximately 300% under light conditions (Figure 4B) [37]. Wang et al. prepared nitrogen-containing compounds by coating CdS NP on *R. palustris* surface-immobilized with N_2_. The accumulated solid biomass (3.44 g/L) reached 2.53 times that of natural cells under visible light radiation [78]. Wang et al. constructed a CdS@*C. beijerinckii* hybrid system and increased the lignocellulosic butanol yield by 23.6% [79]. Ding et al. designed different photocatalytic materials combined with specific functional microorganisms to produce hydrogen and various chemicals, such as IPA, BDO, MKs, H_2_, FA, NH_3_, C_2_H_4_, and PHB [80]. Guo et al. combined InP onto the surface of a genetically engineered yeast strain Δzwf1 (*S. cerevisiae* Δzwf1). They used photogenerated electrons to generate NADPH’s reducing force to promote shikimic acid synthesis inside the cell. The final titer of shikimic acid production reached 48.5 ± 2.1 mg/L [28]. Similarly, Xu et al. hybridized g-C_3_N_4_ with *Ralstonia eutropha* H16, which increased the yield of PHB production of this strain by up to 1.2-fold compared to non-hybridization [71].

### 3.3. Treatment of Environmental Pollution

**Figure 5:**
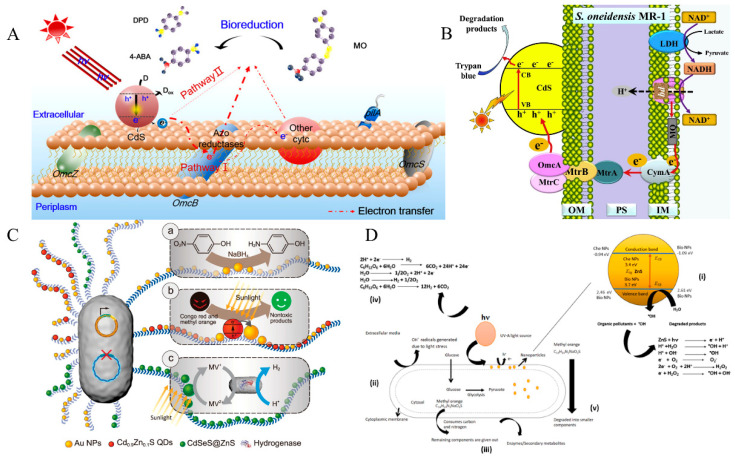
(**A**) Schematic illustration of the degradation pathway of MO using the CdS–*G. sulfurreducens* hybrid system (Copyright © 2019, American Chemical Society). (**B**) *S. oneidensis* MR-1-CdS combines to establish a heterogeneous microbial system for the photocatalytic degradation of trypan blue (© 2019 Published by Elsevier B.V.). (**C**) Diverse catalytic applications of tunable functional *E. coli* biofilms with anchored nano-objects. (**a**) The biofilm-anchored Au NPs enable the recyclable catalytic reduction of the toxic *p*-nitrophenol (PNP) into the harmless *p*-aminophenol (PAP). (**b**) The biofilm-anchored heterogeneous nanostructures (Au NPs/Cd_0.9_Zn_0.1_S QDs) photocatalyze the degradation of organic dyes to low-toxic products based on facile light-induced charge separation. (**c**) The biofilm-anchored quantum dots coupled with the engineered strain enable photoinduced hydrogen production. Electrons are transferred from QDs to hydrogenase using methyl viologen (MV) as a mediator (Copyright © 2019, Oxford University Press). (**D**) Schematic diagram of the photocatalytic mechanism of light-driven *Aspergillus niger* cell-ZnS nano-biohybrids for enhanced removal of the dye methyl orange (Copyright © 1969, Elsevier).

With the rapid growth of industry and agriculture, refractory organic pollutants are released into the environment. By combining the advantages of photocatalysis and bacterial degradation, pollution can be better degraded [81]. Compared with conventional technologies, the photocatalytic material-microorganism hybrid system reduces the energy consumption generated by a high aeration rate and it significantly improves the degradation efficiency. It has developed a new technology with a sufficient reduction power for oxidative pollutants that need to be degraded [82,83]. Xiao et al. constructed a hybrid system under anaerobic conditions. They believed that the holes on the Ag_3_PO_4_ photocatalytic material were removed by the electrons released from *S. oneidensis* MR-1. The electrons generated on Ag_3_PO_4_ were efficiently transferred to rhodamine B, to achieve photoreduction degradation [84]. In their group’s work, a hybrid system was constructed using the photocatalytic material CdS and the microorganism *S. oneidensis* MR-1. Under anaerobic photoexcitation, the electrons generated from the anaerobic respiration of *S. oneidensis* MR-1 act as hole scavengers, and the photocatalytic material CdS continuously generated electrons, thus cleaving the azo bond in the azo dye trypan blue (Figure 5A) [85]. Huang’s team precipitated CdS nanoparticles on the surface of an electrochemically active microorganism (*Geobacter sulfurreducens*) that has two simultaneous degradation pathways. Light-driven CdS nanoparticles generated photogenerated electrons. A part of them was used to reduce the azo dye methyl orange (MO), and microorganisms used the other part for the bioreduction of MO, with 40 mg/L MO able to be wholly degraded within three hours (Figure 5B) [32]. Wang’s group used biofilm-anchored gold nanoclusters alone on the amyloid monomers of genetically modified *E. coli* nanofiber systems to reduce nitrobenzene phenol. Simultaneous anchoring of Cd_0.9_Zn_0.1_S QDs and Au nanoparticles were able to degrade the organic dye Congo Red. The photodegradation rate increased with the increase of Au-QDs volume ratio (Figure 5C) [70]. Priyanka’s group added ZnS nanoparticles to *Aspergillus niger* cells to construct a hybrid system for degrading MO (Figure 5D) [86]. Hybrid systems have a massive potential for treating wastewater that is contaminated with heavy metals. As contaminated sites are generally contaminated with multiple heavy metals, many microorganisms have evolved the ability to tolerate and to detoxify multiple heavy metals simultaneously [81]. Yin et al. proposed a possible reaction where TY3-4 could simultaneously remove Cr^6+^ and Mn^2+^ [82]. If mineral particles with photocatalytic potential are formed to simultaneously mineralize extracellular metal ions, as well as to reduce other metal ions, then degradation can be accomplished without any genetic engineering.

## 4. The Direction of Improvement of the Hybrid System

After construction of the photocatalytic material-microbe hybrid system, the properties of the material and the microorganism itself are affected. This interaction directly affects the electron transfer efficiency. Therefore, improving the charge transfer efficiency is essential for increasing the efficiency of solar energy to chemical energy conversion [75,87,88]. The interaction between materials and microorganisms can be improved in three ways: the nature of the photocatalytic materials, the microorganisms’ properties, and how to composite the two. Through continued interdisciplinary research in biology, chemistry, and physics, achieving net-zero carbon emissions or even harmful carbon emissions in the future becomes possible.

### 4.1. Enhancing the Performances of Photocatalytic Materials

The photocatalytic material can restrict the properties of the photocatalytic material-microbe hybrid system. Under illumination, it is extremely easy for electrons and holes generated on the material surface to compound, which reduces the transformation efficiency of solar energy to chemical energy. It is possible to attenuate the complexation between electrons and holes by using photocatalytic heterojunction materials or by adding sacrificial electron donors [89]. Jiang et al. designed AglnS_2_/In_2_S_3_ heterojunctions to inhibit the complexation of photogenerated electron-hole pairs, improving the utilization of photogenerated electrons in microbial H_2_ production [33]. Gai et al. increased the acetic acid yield of *M. thermoacetica* by designing PDI/PFP p-n heterojunctions to enhance photocatalyst electron-hole pair separation [37].

### 4.2. Improving the Properties of Microorganisms

Along with the evolution of synthetic biology, it is possible to produce the desired chemicals by designing new metabolic pathways or altering existing microbial cellular metabolic pathways. Although extensive genetic manipulation may burden microbial cell metabolism, mild genetic manipulation combined with photocatalytic materials is desirable [90,91]. It can also enhance the yield of microorganisms by promoting electron shuttle secretion and facilitating biofilm formation. Chen’s group engineered the heterotrophic microorganism *Saccharomyces cerevisiae* at multiple levels, increasing the carbon flux into the aromatic amino acid biosynthesis pathway. Guo et al. genetically engineered *Saccharomyces cerevisiae* by knocking out the ZWF1 gene, which encoded glucose-6-phosphate dehydrogenase in the strain, and decoupled the pathway of NADPH production from the central carbon metabolic pathway to maximize carbon flux [28]. Choi et al. genetically modified *Shewanella* strains so that they could use glucose as a carbon and energy source [92].

### 4.3. Improving the Combination of Microbes and Materials

Photocatalytic materials can be complexed with microorganisms to generate energy conversion and catalytic cycling [56]. Different combination methods will produce different interface effects and affect microbial metabolic pathways. In semiconductor material-microbial hybrid systems, the properties of the nanoparticles and microorganisms largely change. Wang et al. genetically engineered *E. coli* so that it could express, secrete, and be sufficient for self-assembly and the anchorage of nanocatalysts outside the cell [70]. Guo et al. used natural polyphenols to modify InP nanoparticles to form modular units that are similar to building blocks, for orderly and tight coupling with cells, thus avoiding the harmful effects of photocatalytic materials on cells [28]. Wei et al. genetically engineered *E. coli* to biologically synthesize biocompatible CdS NPs in situ on the cell surface [30]. In the electrode-microbe hybrid system, meeting the requirements for the size of the photoelectrode reactor, the efficiency of electron transfer across the biomixing interface, and continuous microbial growth are the critical questions that drive the development of the system.

## 5. Summary and Outlook

Currently, the technology for photocatalytic material-microorganism hybrid systems is still in its initial stage. There are still many problems regarding the light energy conversion efficiency, the stability and sustainability of the catalytic system, and the scale of production. In-depth research for improving the system’s temporal stability, biocompatibility, charge transfer efficiency, and energy consumption efficiency is essential to solving the above problems. With the continuous development of materials chemistry and synthetic biology, new photocatalytic materials and microbes that can be applied in the hybrid system will continuously emerge. The selection of suitable photocatalytic materials according to the characteristics of microorganisms, target metabolic pathways, and the surface, composition, structure, and reaction conditions of materials are continuously being optimized. The combination of photocatalytic materials with microbes must affect these microbes. Recently, researchers have found that the number of intracellular proteins and the metabolites of microorganisms changed after binding to photocatalytic materials. The number of substances that are associated with energy metabolism is significantly higher, which is consistent with the logic that photocatalytic materials absorb light energy to supply energy to the cell. However, the intracellular electron transfer pathways are still unknown. The specific electron transfer chain in microorganisms remains to be studied.

In the future, we should further analyze the energy and charge transfer pathways, the intracellular electron transfer chains, and the priority order of energy utilization in heterogeneous systems. This can improve the energy utilization efficiency and then achieve the purpose of efficiently synthesizing the target product. As well as concern for production efficiency, we need to conduct more in-depth studies on cost, stability, and equipment safety, in order to assess the possibility of achieving mass production in the future. The realization of mass production will be a progressive step in the utilization of light energy.

## Figures and Tables

**Table 1:** The typical photocatalytic material-microbe hybrid system.

Build Method	Microorganism	Photocatalytic Material	Composite Method	Function	Efficiency	Ref.
Semiconductor material-microorganism hybrid system	*M. thermoacetica*	CdS NPs	Extracellular deposition	Synthesis acetic acid	1.43 mM per 12 h	[25]
	*M. thermoacetica*	AuNCs	Intracellular suspension	Synthetic acetic acid	6.01 mmol/g per week	[26]
	*C. zofingiensis*	Au NPs	Intracellular suspension	Synthetic carotenoids	10.7 ± 1.2 mg/L	[27]
	*S. cerevisiae* Δ*zwf1*	InP	Extracellular surface modification	Synthetic shikimic acid	48.5 ± 2.1 mg/L	[28]
	*E. coli*	CdS NPs	Extracellular deposition	Hydrogen production	More than 12 µmol/mL/h	[29]
	*E. coli*	CdS NPs	Extracellular surface modification	Hydrogen production	81.80 ± 7.39 μmol per 24 h	[30]
	*E. coli*	I-HTCC	Extracellular surface modification	Hydrogen production	0.71 mM/h	[31]
	*G. sulfurreducens*	CdS	Extracellular deposition	Methyl orange reduction	100% removal rate at 3 h	[32]
	*E. coli*	AglnS_2_/In_2_S_3_	Extracellular surface modification	Hydrogen production	487 µmol/h	[33]
	*Shewanella oneidensis* MR-1	Cu_2_O/RGO	Cell anchoring	Hydrogen production	322.0 μmol/g_Cu2O_ of H_2_ in 4 h	[34]
	*C. pyrenoidosa*	PBF	Extracellular surface modification	Promote O_2_, NADPH, and ATP synthesis	Respectively, up 120%, 97%, and 76%	[35]
Dye/polymer-microbial hybrid system	*Synechococcus* sp. PCC7942, *Syne*	PFP	Extracellular surface modification	Promote O_2_, NADPH, and ATP synthesis	Respectively, up 52.8%, 47.9%, and 27.2%.	[36]
	*M. thermoacetica*	PFP/PDI	Extracellular surface modification	Synthetic acetic acid	Accumulated 0.63 mM in 3 days	[37]
	*C. pyrenoidosa*	PPE	In situ modification of cell surface	Synthesize ATP	500 μM monomer improved 115% after 30 min of light	[38]
	*S. ovata*	Silicon nanowire	Integrated combination	Synthetic acetic acid	1200 mg/L/d	[39]
Electrode-Microbial Hybrid System	*S. ovata*	Silicon nanowire	Integrated combination	Synthetic acetic acid	0.3 g/L/d	[40]
	*X. autotrophicus*	CoP-CoPi	Distributed combination	Synthetic biomass	To 6.2% biomass in 24 h	[41]
	*R. eutropha*	CoPi-NiMoZn	Distributed combination	Synthetic biomass	Up to 216 ± 17 mg/L	[42]
	*R. eutropha*	Co-P alloy-CoPi	Distributed combination	Synthetic biomass and fusel alcohols	CO_2_ reduction energy efficiency of ~50%	[43]
	*S. ovata*	CoP-CoPi	Distributed combination	Synthetic acetic acid	1.1 mM/h	[44]
	Methanogens	TiO_2_/CdS	Distributed combination	Synthesis of CH_4_	1925 mL/m^2^/d	[45]

## Data Availability

Data sharing not applicable. No new data were created or analyzed in this study.
